# Optimal cutoff for the evaluation of insulin resistance through triglyceride-glucose index: A cross-sectional study in a Venezuelan population

**DOI:** 10.12688/f1000research.12170.3

**Published:** 2018-01-30

**Authors:** Juan Salazar, Valmore Bermúdez, María Calvo, Luis Carlos Olivar, Eliana Luzardo, Carla Navarro, Heysa Mencia, María Martínez, José Rivas-Ríos, Sandra Wilches-Durán, Marcos Cerda, Modesto Graterol, Rosemily Graterol, Carlos Garicano, Juan Hernández, Joselyn Rojas

**Affiliations:** 1 Endocrine-Metabolic Research Center, , University of Zulia, Maracaibo, Venezuela; 2 Grupo de Investigación Altos Estudios de Frontera (ALEF), Universidad Simón Bolívar, Cúcuta, Colombia; 3 Division of Pulmonary and Critical Care Medicine, Brigham and Women’s Hospital and Harvard Medical School, Boston, MA, 02115, USA

**Keywords:** insulin resistance, triglycerides, glycemia, Metabolic syndrome, cutoff

## Abstract

**Background:** Insulin resistance (IR) evaluation is a fundamental goal in clinical and epidemiological research. However, the most widely used methods are difficult to apply to populations with low incomes. The triglyceride-glucose index (TGI) emerges as an alternative to use in daily clinical practice. Therefore the objective of this study was to determine an optimal cutoff point for the TGI in an adult population from Maracaibo, Venezuela.

**Methods:** This is a sub-study of Maracaibo City Metabolic Syndrome Prevalence Study, a descriptive, cross-sectional study with random and multi-stage sampling. For this analysis, 2004 individuals of both genders ≥18 years old with basal insulin determination and triglycerides < 500 mg/dl were evaluated.. A reference population was selected according to clinical and metabolic criteria to plot ROC Curves specific for gender and age groups to determine the optimal cutoff point according to sensitivity and specificity.The TGI was calculated according to the equation: ln [Fasting triglyceride (mg / dl) x Fasting glucose (mg / dl)] / 2.

**Results: **The TGI in the general population was 4.6±0.3 (male: 4.66±0.34 vs. female: 4.56±0.33, p=8.93x10 ^-10^). The optimal cutoff point was 4.49, with a sensitivity of 82.6% and specificity of 82.1% (AUC=0.889, 95% CI: 0.854-0.924). There were no significant differences in the predictive capacity of the index when evaluated according to gender and age groups. Those individuals with TGI≥4.5 had higher HOMA2-IR averages than those with TGI <4.5 (2.48 vs 1.74, respectively, p<0.001).

**Conclusions: **The TGI is a measure of interest to identify IR in the general population. We propose a single cutoff point of 4.5 to classify individuals with IR. Future studies should evaluate the predictive capacity of this index to determine atypical metabolic phenotypes, type 2 diabetes mellitus and even cardiovascular risk in our population.

## Introduction

Insulin resistance (IR) is a metabolic condition in which insulin-dependent tissues become less sensitive to insulin action, leading to an imbalance in the metabolism of carbohydrates, lipids and proteins ^1^. This condition is caused by the influence of different risk factors in the population, such as aging, alcohol consumption, smoking, hypercaloric diets, sedentary lifestyle and obesity ^2^. Its role in the development of different pathologies, as cardiovascular disease (CVD) ^3^ and cerebrovascular disease ^4^, is now recognized, as well as playing an important role in the pathogenesis and clinical outcomes of the metabolic syndrome (MS) ^5^ and type 2 diabetes mellitus (DM2) ^6^.

In 1963, Randle and colleagues were among the first to investigate the pathophysiology of IR ^7^, suggesting the elevation of free fatty acids (FFA) in the splenic circulation as the cornerstone of this disorder. They proposed the glucose fatty acid cycle, called the Randle cycle. Years later, a theory proposed by Shulman ^8^ surfaced, which continued to support the role of FFA in the pathophysiology of IR. However, these authors suggested that FFA and its products, such as diacylglycerol, acyl-coA and ceramides, activate serin-threonine kinases, which phosphorylate important proteins, inhibit the insulin signaling pathway and subsequently translocated glucose transporter 4 (GLUT4) to the plasma membrane ^8^.

Although many aspects remain to be clarified in the pathophysiology of IR, its long-term complications have generated great interest in the determination of ideal methods that allow the promotion of an early and accurate diagnosis in risky populations ^9^. In this sense, the Euglycemic-Hyperinsulinemic Clamp is considered the gold standard for the determination of IR ^10^, but the high cost and impracticability of this method has promoted the development of new techniques for the estimation of insulin sensitivity. Many mathematical models have been proposed in recent years with the objective of simplifying the measurement of IR ^11^, highlighting the Homeostasis Model Assessment (HOMA-IR), a validated method to measure IR from serum glucose and fasting serum insulin ^12^. This index has been studied in our population, which was conducted in 2026 subjects and evaluated the factors related to insulin resistance (defined as HOMA2-IR=2) ^13^. However, one of the biggest limitations is the lack of accessibility to populations with lower incomes, since all the individuals require insulin blood tests.

Simental-Mendia *et al*. ^14, 15^ have proposed and validated a new formula to evaluate IR from the levels of serum triglycerides (TAG) and fasting glucose (FG), which is known as ‘Triglyceride/Glucose Index’ (TGI), this formula is a potential diagnostic tool when other standard methods are not available. Based on the information above, the objective of the study is to determine an optimal cut point of the TGI to determine IR and to evaluate the behavior according to the main sociodemographic characteristics in an adult population from Maracaibo, Zulia, Venezuela.

## Methods

### Sample selection and study design

The Maracaibo City Metabolic Syndrome Prevalence Study (MMSPS) study is a descriptive, cross-sectional, randomized, multi-stage sampling study that was carried out in Maracaibo-Venezuela; the second most populated city in the country with an approximate population of 2,500,000, in order to evaluate the cardiovascular and metabolic risk factors of this locality, during the period May 2007 - December 2009 ^16^. The only inclusion criteria was individuals older than 18 years. The sample (1,986 individuals) was calculated based on the estimates of the population in the city given by the National Institute of Statistics (1,428,043 inhabitants for 2007). A total of 244 individuals (12%) were added through oversampling, in order to increase the accuracy of the estimates obtained from the smaller subgroups of the sample, representing a total of 2230 individuals of both genders. Sampling details have been previously published ^16^. The study was approved by the Bioethics Committee of the Endocrine and Metabolic Research Center – University of Zulia (approval number: BEC-006-0305). This ethical approval included all future studies that used the data from the MMSPS. All participants signed written consent before being questioned and physically examined by a trained team.

**Figure 1.**
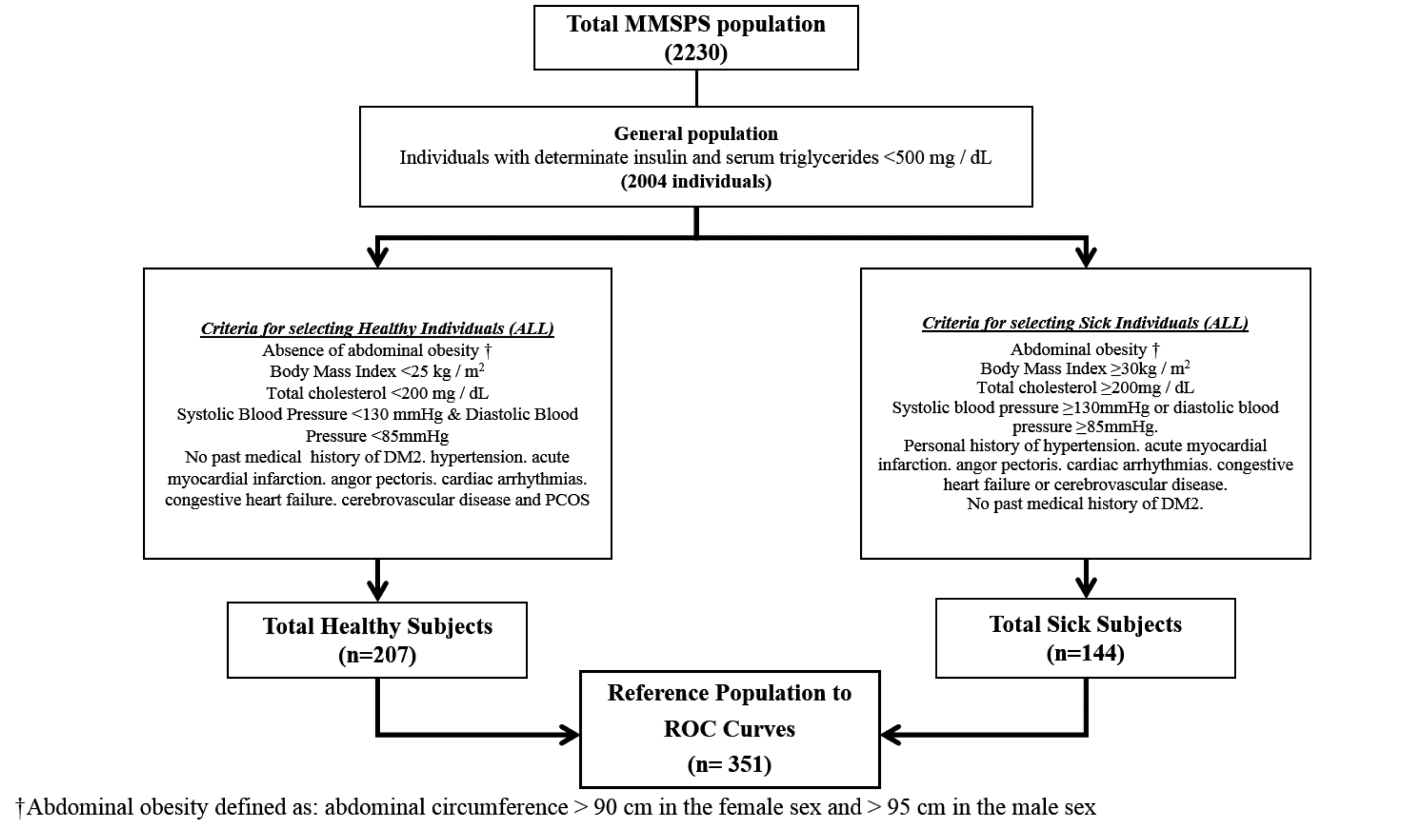
Diagram of reference population selection for cut-off points determination of the triglyceride-glucose index in Maracaibo city, Venezuela. ROC Curves: Receiver operating characteristic curves.

For this analysis, 2004 individuals were selected according to the availability of baseline insulin, in addition to the exclusion of individuals with TAG≥500 mg/dl. Based on this sub-sample, a reference population was selected based on all the following criteria: abdominal obesity, total cholesterol, high blood pressure and personal history of DM2, coronary artery diseases, cardiac arrhythmias, acute cerebrovascular disease, and polycystic ovaries; with the purpose of establishing a subsample of healthy and unhealthy subjects without using definitions or diagnostic criteria that include TAG and glycaemia values to avoid variables correlation *a priori* ( Figure 1).

### Individual evaluation

The data was gathered through a complete medical history performed by the trained team: the past medical and family history for cardiovascular and endocrine-metabolic diseases was assessed.

### Blood pressure and anthropometric evaluation

The auscultation method was used for the measurement of blood pressure, using stethoscopes and calibrated sphygmomanometers adequately validated. The procedure was performed with the individual at rest (at least 15 minutes) and sitting with both feet on the floor; three measurements were taken, with 15 minutes of separation between one measurement and the other. Systolic blood pressure was determined by auscultation of the first Korotkoff noise, while diastolic blood pressure was determined on auscultation of the fifth Korotkoff noise.

Weight was determined through a dielectric balance (Tanita, TBF-310 GS Body Composition Analyzer, Tokyo, Japan), and height was obtained by using vertical tape calibrated in centimeters and millimeters. Individuals were standing and barefoot, with light clothing throughout the evaluation, maintaining a straight posture and head up. Body Mass Index was calculated using the weight/height formula and classified according to the criteria proposed by the World Health Organisation (low weight, normal weight, overweight, Obesity type I, II and III) ^17^. The measurement of the abdominal circumference was taken with a plastic metric tape in centimeters at equidistant points between the costal ridge and the iliac crest, according to the protocol proposed by the National Salute Institute of the United States ^18^. Abdominal obesity was defined according to specific cutoff points for our previously determined population, ≥90 cm in women and ≥95 cm in men ^19^.

### Laboratory analysis

Blood samples were collected between 8:00 A.M. and 10:00 A.M. after an overnight fast (~10 hours). Determination of glucose, total cholesterol, triglycerides, and HDL-C was done with an automated analyzer (Human Gesellschaft fur Biochemica und Diagnostica mbH, Germany). The intra-assay coefficient of variation for total cholesterol, TAG, glucose and insulin was 3%, 5%, 3%, and <10%, respectively. Insulin was determined using an ultrasensitive ELISA double-sandwich method (DRG Instruments GmbH, Germany, Inc.), with a Detection Limit <1 mU/L. The HOMA2IR index was calculated using the software administered by the Oxford Diabetes Center, Endocrinology and Metabolism (available at http://www.dtu.ox.ac.uk/homacalculator/index.php). A cutoff value of ≥2 was used to determine IR ^20^.

### Calculation of TGI

The calculation of the TGI was done using the equation: ln [Fasting TAG (mg/dl) x FG (mg/dl)]/2; thus being expressed on a logarithmic scale ^14, 21^.

### Statistical analysis

Qualitative variables were expressed in absolute and relative frequencies. While the quantitative variables were expressed as arithmetic mean ± SD, with the previous analysis of normality through means of a Geary test. Significant differences between groups were assessed using the Student’s *t*-test, while an ANOVA was used for comparisons between three or more groups. Data were analyzed through the SPSS v.21 for Windows (IBM Chicago, IL), considering statistically significant results when p <0.05.

ROC curves were plotted in the reference population ( Figure 1) to analyze the predictive capacity and to determine an optimal cutoff point for the TGI. ROC curves were gender-specific using R version 3.4.1. Several indices were calculated to evaluate the optimum cutoff point in the curve. The area under the curve (AUC) is used to establish the ability of the test to obtain an appropriate cutoff where an AUC of 1.00 is considered a perfect diagnostic test ^22^. Comparisons between AUC were performed using Delong’s test ^23^. The Youden Index (J) was calculated using the formula [J = sensitivity + specificity-1 = S-(1-Es)] ^23^, obtaining the value of true positives (sensitivity) and false positives (1-specificity) when J >1. The minimum cutoff point was calculated using the nearest point to 0.1 in the ROC curves formula: square root of [(1-sensitivity) ^2^ (1-specificity) ^2^] ^24^. In addition, probability radius positive [sensitivity/1-specificity] and negative [1-sensitivity/specificity] were calculated to aid in the selection of the cutoff point together with the Youden index. Likelihood values >1 indicate association with the disease, while those <1 indicate association with the absence of the disease ^25^.

## Results

### General characteristics of the sample

A total of 2004 individuals were studied, 53.4% (n = 1050) were female, the mean age of the population was 39.6±15.3. The general characteristics of the population are shown in Table 1. The mean TGI in the general population was 4.6±0.3, with higher values in males (males: 4.66±0.34 vs. females: 4.56±0.33, p=8.93×10 ^-10^). The epidemiological behavior of the TGI according to age and ethnicity is shown in Table 2, which shows an increase of the index as age increases. In regards to ethnicity, no statistically significant differences were found between means (p=0.326).

**Table 1.** General characteristics of the sample studied, Maracaibo city, Venezuela.

	Female (n= 1050)		Male (n=954)		Total (n=2004)	
	n	%	n	%	n	%
**Age (years)**						
<30	308	29.3	363	38.1	671	33.5
30–49	420	40.0	350	36.7	770	38.4
≥50	322	30.7	241	25.3	563	28.1
**Ethnicity**						
Mixed	794	75.6	740	77.6	1534	76.5
White Hispanic	171	16.3	145	15.2	316	15.8
Afro-Venezuelan	27	2.6	32	3.4	59	2.9
American Indian	47	4.5	36	3.8	83	4.1
Other *	11	1.0	1	0.1	12	0.6
**Triglycerides** **(mg/dl)**						
<150	815	77.6	644	67.5	1459	72.8
≥150	235	22.4	310	32.5	545	27.2
**Glycaemic** **status ^¶^**						
Euglycemic	774	73.9	662	69.4	1436	71.7
Impaired fasting glucose	186	17.7	212	22.2	398	19.9
Type 2 diabetes mellitus	88	8.4	80	8.4	168	8.4

*Asian and Arabic descent. ^¶^Criteria according to the ADA 2016 consensus

**Table 2.** Epidemiological behavior of the triglyceride-glucose index in the general population according to sociodemographic variables, in Maracaibo city, Venezuela.

	n	TGI (n=2004)	
		Mean±SD	p ^*^
**Gender**			8.93×10 ^−10^
Female	1050	4.56±0.33	
Male	954	4.66±0.34	
**Age (years)**			6.21×10 ^−77^
<30	671	4.43±0.28	
30–49	770	4.65±0.33	
≥50	563	4.77±0.31	
**Ethnicity**			0.326
Mixed	1534	4.60±0.33	
White Hispanic	316	4.62±0.35	
Afro-Venezuelan	59	4.68±0.32	
American Indian	83	4.60±0.31	
Other ^¶^	12	4.51±0.28	

* Student’s *t*- test (for more than two groups one-way ANOVA was used). ¶ Asian and Arabic descent. SD: standard deviation Post-hoc Tukey: <30 years vs 30–49 years, p=5.09x10 ^−9^; <30 years vs ≥50 years, p=5.09x10 ^−9^; 30–49 years vs ≥50 years, p=5.10x10 ^−9^.

When assessing the reference population (n=351); healthy: n=207 – unhealthy: n=144, a similar behavior of the TGI was found according to age and ethnicity ( Table 3). In addition, there were no differences in TGI mean between the general and reference population.

**Table 3.** Epidemiological behavior of the triglyceride-glucose index in the reference population according to sociodemographic variables, in Maracaibo city, Venezuela.

	TGI (n=351)		
	**n**	**Mean±SD**	p *
**Gender**			5.83-10 ^-6^
Female	190	4.41±0.29	
Male	161	4.56±0.32	
**Age (years)**			8.42×10 ^-28^
<30	178	4.31±0.29	
30–49	118	4.61±0.31	
≥50	55	4.72±0.23	
**Ethnicity**			0.413
Mixed	268	4.48±0.32	
White Hispanic	52	4.48±0.34	
Afro-Venezuelan	8	4.59±0.26	
American Indian	20	4.39±0.23	
Other ^¶^	3	4.25±0.26	

*Student’s *t-*test (for more than two groups one way-ANOVA was used). ¶ Asian and Arabic descent. SD: Standard deviation. Post-hoc Tukey: <30 years vs 30–49 years, p=5.09×10 ^-9^; <30 years vs ≥50 years, p=5.09x10 ^-9^; 30–49 years vs ≥50 years, p=0.03.

### Cutoff points for the TGI in the reference population by gender

**Figure 2.**
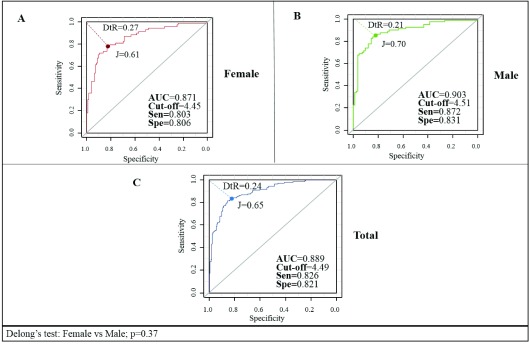
Receiver Operating Characteristic curves for the triglyceride-glucose index in the reference population by gender, in Maracaibo city, Venezuela. DtR, Distance to ROC; J, Youden Index; AUC, are under the curve; sen, sensitivity; spe, specificity.

For the determination of cutoff points of the TGI, ROC curves were plotted for the reference population by gender ( Figure 2). An AUC of 0.889 (95% CI: 0.854-0.924) was obtained for the general population with a proposed cutoff of 4.49 (82.6% sensitivity, and 82.1% specificity), while the AUC calculated for males was 0.903 (95% CI: 0.856-0.950) and for females was 0.871 (95% CI: 0.818-0.925); Delong's test: p=0.37. The cutoff points and index calculated according to the ROC curves are shown in Table 4.

**Table 4.** Cut-off points for triglyceride-glucose index (TGI) selected in the reference population and by gender, in Maracaibo city, Venezuela.

Gender	TGI ^¶^	Sensitivity (%)	Specificity (%)	Youden index	ROC distance	LR+	AUC (95%CI)
**Female**	4.45	80.3	80.6	0.61	0.276	4.14	0.871 (0.818–0.925)
**Male**	4.51	87.2	83.1	0.70	0.212	5.15	0.903 (0.856–0.950)
**Total**	4.49	82.6	82.1	0.65	0.249	4.61	0.889 (0.854–0.924)

^¶^Cut-off points selected according to the best combination of indices. Delong’s test= 0.37

### Cutoff points for the TGI in the reference population by age

When assessing the predictive capacity of the TGI according to age, a higher AUC was obtained in individuals between the ages of 30 and 50 years old (AUC=0.876; 95% CI: 0.812-0.939); however, when comparing AUCs among age groups, no significant differences were found. The cutoff points obtained according to age are shown in Table 5, with means similar to that proposed for the reference population (<30 years: 4.48 [65.0% sensitivity, and 84.8% specificity]; 30–50 years: 4.51 [84.7% sensitivity, and 78.3% specificity], ≥50 years, 4.51 [86.5% sensitivity, and 66.7% specificity]).

**Table 5.** Cutoff points for triglyceride-glucose index (TGI) selected in the reference population according to age groups, in Maracaibo city, Venezuela.

Age (years)	TGI ^¶^	Sensitivity (%)	Specificity (%)	Youden index	ROC distance	LR+	AUC (95%CI)
**<30**	4.49	65.0	84.8	0.50	0.381	4.27	0.789 (0.684–0.895)
**30–50**	4.51	84.7	78.3	0.63	0.265	3.89	0.876 (0.812–0.939)
**≥50**	4.51	86.5	66.7	0.53	0.359	2.59	0.776 (0.602–0.949)

^¶^Cut-off points selected according to the best combination of indices. Delong’s test: <30 vs 30–50 years; p=0.171. <30 vs >50 years; p=0.885. 30–50 vs >50 years; p=0.34.

### HOMA2IR according to cutoff points of the TGI

**Figure 3.**
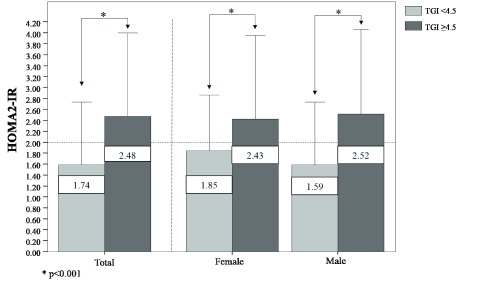
HOMA2-IR levels according to the specific cutoff point for the triglyceride-glucose index in the general population, in Maracaibo city, Venezuela.

Finally, when assessing HOMA2-IR levels according to the proposed cutoff point of the TGI for the general population ( Figure 3), individuals with TGI≥4.5 exhibited higher levels of HOMA2IR than those who had a TGI <4.5 (2.48 vs 1.74, respectively, p<0.001), with similar behavior by gender.

Data for the study ‘Optimal cutoff for the evaluation of insulin resistance through triglyceride-glucose index: A cross-sectional study in a Venezuelan population’ This data is available in both .SAV and .xls forms. BMI: Body Mass Index; BP: Blood Pressure. Click here for additional data file. Copyright: © 2018 Salazar J et al. 2018 Data associated with the article are available under the terms of the Creative Commons Zero "No rights reserved" data waiver (CC0 1.0 Public domain dedication).

## Discussion

The evaluation of IR is an objective that continues to acquire relevance in clinical and epidemiological research, due to the potential role of this disorder in the pathophysiology of MS ^26^, the consequent risk of developing DM2 ^27^ and CVD ^28^. In developing countries with economic difficulties in health systems, such as Venezuela, routine measurements of plasma insulin are not easily accessible, which forces the use of other indices based on the role of glucolipotoxicity as a key element in the development of IR ^29^. In this way, the TGI has recently been proposed and validated as a useful alternative in clinical settings, and has been well accepted due to the high predictive power observed with respect to other indices ^30^.

Several studies have shown that the TGI better predicts HOMA-IR levels than variables, such as the TAG/HDL index, visceral adiposity index, leptin, Apo-B/Apo-AI, and lipid parameters ^29, 30^, representing a very good correspondence with this index ^31^, which constitutes an important tool with high validity for the clinician when facing limited access to lab work-ups. However, in view of the wide variability of TAG levels according to the ethnic, sociocultural and genetic characteristics of each population, the need arises to evaluate their epidemiological behavior and establish reference values specific to each region.

In regards to our study, the mean TGI was higher in men compared to women. Several studies have reported similar findings ^14, 32^; however, when plotted ROC curves for the TGI for each gender, significant differences in the AUC values and effect size by gender were not observed. We propose the use of a single cutoff point of 4.5 for the identification of unhealthy individuals in the clinical practice as an easy tool for the clinician.

These findings corresponded to those originally reported by the index’s authors in 2010 ^15^, when evaluating the discriminative ability of this index to determine IR against the euglycemic-hyperinsulinemic clamp in a population of 99 individuals (11 healthy, 34 obese, 22 with prediabetes and 32 with DM2) suggesting a cutoff point of 4.68 with high sensitivity (96.5%) and specificity (85.0%). Based on these findings, several studies have attempted to establish specific cutoff points for their populations, assessing the clinical utility of this index. In 2011, in a Brazilian population, Vasques *et al*. ^33^ stipulated that the TGI had a slightly better performance to diagnose IR compared to HOMA2-IR (AUC=0.79 vs AUC=0.77, respectively). Although these investigators did not perform any statistical tests to compare the diagnostic capacity between both indices, the similar behavior allows the use of TGI in clinical settings routinely.

On the other hand, Unger *et al*. ^21^ conducted a cross-sectional study in an Argentinean population with the aim of evaluating the discriminative capacity of the TGI for the diagnosis of MS, taking into account that the development of this syndrome is related to IR. They set a cutoff point of 8.8 (sensitivity = 79%, specificity = 86%) to diagnose MS in their population, a value that differs markedly from the values in our region and those originally proposed. These differences have been observed in other studies ^34– 36^, so considering that the means of TAG do not vary significantly between these and our study, this discrepancy could be attributable to errors in the calculation of the original formula ^37^.

In regards to age, an increase in the average TGI was observed as age increased, similar to that found by Navarro-González *et al.* ^38^ and Cuda *et al*. ^39^. This can be associated with the increase in oxidative stress inherent to aging, which would favor the development of IR, as well as the elevation of plasma levels of TAG ^40^. In this sense, Guerrero-Romero *et al*. ^31^ recently evaluated the performance of the TGI to determine IR against the euglycemic-hyperinsulinemic clamp in a young Mexican adult population (mean age: 19.2±1.4), with the goal of establishing a specific cutoff point in this population. However, the cutoff points of 4.55 in men and 4.68 in women were similar to the value proposed previously for the general population, and similar to the one proposed in our study. Also, when we plotted ROC curves for each age group, we did not observe significant differences between AUC neither effect size in Cohen’s *d* analysis, ruling out the need to establish age-specific cutoff points.

In relation to ethnicity, the small sample in each of the categories precludes the generalization of these results, but it shows that it is necessary to evaluate the behavior of the TGI in the different ethnic groups on a large scale and to determine if there are significant differences between them because there is no data reported on this diagnostic method among various ethnicities.

Base on previous analyzes, the usefulness of the TGI has been extended in several regions of the world, being also used in the screening of other pathological metabolic states in which the IR underlies the fundamental pathophysiological mechanism, such as DM2 ^41^ and atypical metabolic phenotypes ^42, 43^. In fact, the TGI appears to be a better predictor of incidence of DM2 than TAG ^38^, weight gain ^43^ and other IR indices, such as TAG/HDL and HOMA-IR ^41^. Moreover, Lee *et al*. performed a retrospective study involving 2900 Korean non-diabetic adults determinate as a cutoff point 8, (AUC=0.751, 95% CI 0.704-0.799) to predict the incidence of DM2 in their population. In addition, only individuals with a TGI >8.8 were associated with a significant risk of DM2 incidence, regardless of the presence of obesity ^44^.

Additionally, the index can better predict the patient's metabolic status, as it has been shown in discriminating atypical metabolic phenotypes, such as metabolically obese normal weight and healthy obese, as well as their progression throughout time ^35^. Cutoff points have been determined to discriminate these pathological metabolic status in two populations of Korea ^34, 35^, which could facilitate the definition of these phenotypes. Future studies in our population should evaluate the utility of this index in the identification of these abnormal pathological statuses, considering an appropriate inference when used in subjects with extremely high triglyceride levels. It is important to mention that within the limitations of this study it is necessary to consider the cross-sectional design and the lack of gold standard during the process of selection of the reference population, thus is difficult to evaluate a direct causal relationship. Likewise, this analysis is limited to a Latin-American population, extrapolating the findings to other ethnicities should be interpreted cautiously; there might be a need for ethnic specific TGI cut-offs, as Korean, Italian and Chinese population have made.

Additionally, there are several factors that can influence the TGI values and therefore cutoff points selection; these include lipid-lowering medications, nutritional and physical activity variables which are able to regulate the metabolism of equation components and be potential strategies in the management of IR. However, only the use of lipid-lowering drugs was considered in the selection of the reference population, excluding those subjects who had a regular consumption of statins or fibrates, the role of diet and physical activity will be considered in future studies that determine the effect of environmental components in a multivariate context.

## Conclusions

The TGI is an instrument of interest when it comes to identifying IR in the general population. We propose a single cutoff point of 4.5 to identify patients with IR, as we identify the need for standardization of the formula calculation in order to be able to adequately compare the differences observed in various studies. Despite this, due to the easier application in clinical practice, future studies in our population should evaluate the predictive capacity of this index to determine atypical metabolic phenotypes, DM2 and CVD risk.

## Data availability

The data referenced by this article are under copyright with the following copyright statement: Copyright: © 2018 Salazar J et al.

Data associated with the article are available under the terms of the Creative Commons Zero "No rights reserved" data waiver (CC0 1.0 Public domain dedication). 

Dataset 1: Data for the study ‘Optimal cutoff for the evaluation of insulin resistance through triglyceride-glucose index: A cross-sectional study in a Venezuelan population’. This data is available in both .SAV and .xls forms. BMI: Body Mass Index; BP: Blood Pressure. doi, 10.5256/f1000research.12170.d171840 ^45^
