# Supplementary material for: Optimal cutoff for the evaluation of insulin resistance through triglyceride-glucose index: A cross-sectional study in a Venezuelan population
Source: F1000Res. 2018 Jan 30;6:1337. Originally published 2017 Aug 7. [Version 3] doi: 10.12688/f1000research.12170.3 (PMC5760971; doi:10.12688/f1000research.12170.3)
Supplement: Peer review history

# Peer review: Referee response for version 2

**DOI:** 10.5256/f1000research.14039.r28438

**Published:** 2018-01-05

**Authors:** Muthuswamy Balasubramanyam

**Affiliations:** 1 Department of Cell and Molecular Biology, Madras Diabetes Research Foundation (MDRF), Chennai, India

Insulin resistance (IR) is the root cause for several major metabolic disorders and identifying individuals with IR is right now a public health priority as this will pave way for appropriate prevention and management of type 2 diabetes and associated non-communicable diseases. While Euglycemic-Hyperinsulinemic Clamp which is considered as the gold standard for the determination of IR is an invasive and impractical procedure for risk assessment in the general population, the measurement of IR by Homeostasis Model Assessment (HOMA-IR) also requires costly tests like insulin assays which are difficult in the low-resource settings. In this context, assessing IR and metabolic risk via triglyceride-glucose index (TGI) as detailed in this manuscript receives some attention and importance. The main advantages of the TGI is that both the triglyceride and glucose measurements are cost-effective and hence affordable in epidemiological and screening programs to assess the relative cardiometabolic risk in the general populations and in the clinics.

 However, the manuscript should delineate several limitations of the study (as detailed below):

- As the study nature is of cross-sectional observation study design, it is difficult to evaluate a direct causal relationship.
- TGI may have limited inference when used in subjects with extremely high triglyceride levels.
- Extrapolating the findings of this study to other races or ethnicities should be interpreted cautiously; there might be a need for ethnic / race specific TGI cut-offs.
- TGI may suffer from confounding factors such as medications that lower mainly triglycerides. Authors should discuss these medication (statins, fenofibrate, omega-3 etc) effects.
- Also, TGI values could be confounded by diet and physical activity among the groups. Therefore, authors should discuss the role of diet (if FFQ is available) and physical activity in relation to cut-offs of TGI. These should be discussed.

I have read this submission. I believe that I have an appropriate level of expertise to confirm that it is of an acceptable scientific standard.

# Peer review: Referee response for version 1

**DOI:** 10.5256/f1000research.13172.r25043

**Published:** 2017-08-25

**Authors:** Silvia Fabiana Benozzi, Gisela Unger

**Affiliations:** 1 Universidad Nacional del Sur, Bahía Blanca, Argentina

This research article deals with an interesting subject related to insulin resistance, its evaluation without the need of an insulin blood test. We believe that it can contribute to identify patients with metabolic risk. Authors have clearly elaborated the background of the problem and current literature is cited. The study is properly conducted and described. But, there are some shortcomings that should be corrected:

- Abstract: Line 1, there are two dots after the word *evaluated*.
- Keywords are absent.
- Abbreviations: Page 3, we considered unnecessary this subtitle because the abbreviations are spelled out in the text.
- Sample selection and study design: We have interpreted that the presence of all the cited criteria was necessary to classify an individual in the reference population, but it is not clear in the text. Authors should explain how many of the cited criteria were considered to classify an individual as healthy or sick.
- Figure 1: Authors should consider the previous correction also in this figure. Authors should replace the explanation *^†^Abdominal circumference > 90 cm in the female sex and > 95 cm in the male secx *with *^† ^Abdominal obesity defined as: abdominal circumference > 90 cm in the female sex and > 95 cm in the male sex*. Authors should highlight in the respective box that the population of 2004 individuals is the General population.
- Laboratory analysis: If possible, authors should provide the fasting time in hours owing the lack of harmonization in this preanalytical aspect worldwide. Glucose and insulin analytical coefficients of variation should be provided. It is not necessary HDL-C description as this analyte was not used in the present research.
- Statistical analysis: Authors should verify the ROC curve formula described to calculate the minimum cutoff point.
- Results: Authors should correct the second subtitle because the cutoff points were calculated in the reference population (n=351) – as stated in Methods – and not in the general population (n=2004); also in the second line of the respective text. Authors should correct the third subtitle because the cutoff points were calculated in the reference population (n=351) – as stated in Methods – and not in the general population (n= 2004); authors should clarify the comparison made in line 7 of the respective text, we understand that they are comparing the cutoff values obtained by age with the cutoff value obtained for the total reference population.
- Table 2: Authors should replace the word *TOTAL* with *TGI* so as to allow a better interpretation of the values shown in the table. Also the number of individuals should be described by variable, as in Table 3.
- Table 3: Authors should replace the word *TOTAL* with *TGI* so as to allow a better interpretation of the values shown in the table.
- Table 4: Authors should correct the table title because the cutoff points were calculated in the reference population (n=351) – as stated in Methods – and not in the general population (n= 2004).
- Table 5: Authors should indicate in the title that the cutoff points were calculated in the reference population (n= 351),as in the other tables.
- Figure 2: Authors should correct the title because the cutoff points were calculated in the reference population (n=351) – as stated in Methods – and not in the general population (n= 2004).
- Figures and tables in general: Authors should verify that each figure or table has a concise title of no more than 15 words. Authors should provide a legend for each figure and table that briefly describes the key points and explain any symbols and abbreviations used. Also the *n* value should be provided in each table and figure to help reader’s interpretation.
- Discussion: Authors states that TGI was higher in men compared to women, and that an increase in its average value was observed as age increased. It would be interesting to evaluate the effect size of these statistical differences so as to interpret the clinical importance of these increases;Cohen’s *d*─https://www.uccs.edu/~lbecker/─ can be used for this purpose if the variable has a parametric distribution.
- General considerations: Authors should express *p* values with three decimals in all the text, tables and figures. The word *cutoff* is written as *cut-off* in same parts of the text, figures and tables, the same happens with the word *indexes* and *indices. *They should be written in the same way in all the text. It would be advisable to replace in the introduction: *blood work to calculate the serum insulin *with *insulinblood test*; in all the text: *sick* with *unhealthy*; in the conclusions: *generalization* with *harmonization* or *standardization*.

We have read this submission. We believe that we have an appropriate level of expertise to confirm that it is of an acceptable scientific standard.
